# Diagnostic Accuracy and Clinical Impact of Handheld Point-of-Care Ultrasound in Pediatric Odontogenic Infections: A Prospective Cohort Study

**DOI:** 10.3390/children12101392

**Published:** 2025-10-15

**Authors:** Hanna Frid, Amir Bilder, Ahmad Hija, Omri Emodi

**Affiliations:** 1 Oral and Maxillofacial Surgery, Rambam Medical Care Campus, HaAliya HaShniya St 8, Haifa 3109601, Israel; h_frid@rambam.health.org.il (H.F.); a_bilder@rambam.health.gov.il (A.B.); o_emodi@rambam.health.gov.il (O.E.); 2 Ruth & Bruce Rappaport Faculty of Medicine, Technion–Israel Institute of Technology, Haifa 3109601, Israel

**Keywords:** ultrasound, abscess, odontogenic infection, pediatric admission, Emergency Department (ED), Point-of-Care Ultrasound (POCUS), Surgical intervention, handheld ultrasound, diagnostic accuracy

## Abstract

Background: Pediatric odontogenic infections pose significant diagnostic challenges, particularly in distinguishing between cellulitis and abscess. Accurate differentiation is crucial for guiding appropriate management—antibiotics alone for cellulitis versus surgical incision and drainage (I&D) for an abscess—but can be difficult without specialized expertise or advanced imaging. Objective: We aimed to evaluate the diagnostic accuracy of handheld point-of-care ultrasound (POCUS; Philips Lumify), utilized by non-specialist clinicians, in differentiating cellulitis from abscess in pediatric odontogenic infections. A secondary objective was to assess its impact on reducing hospital admissions and emergency department (ED) burden. Methods: This prospective cohort study involved 111 pediatric patients (aged 1–17 years) presenting with maxillofacial odontogenic infections to a tertiary care academic medical center. Following clinical evaluations, handheld POCUS assessments were performed by trained non-specialist clinicians. Findings from I&D or clinical resolution with antibiotics served as the reference standard. Ninety cases were included in the final diagnostic accuracy analysis after 21 exclusions. Sensitivity, specificity, positive predictive value (PPV), negative predictive value (NPV), and diagnostic accuracy with 95% confidence intervals (CIs) were calculated. Hospital admission trends were compared before (2017–2021) and after POCUS implementation (January 2022–April 2025). Interpretation should consider potential verification bias from the asymmetric reference standard (I&D for abscess vs. clinical resolution for cellulitis). Results: Handheld POCUS exhibited a sensitivity of 72.97% (95% CI: 57.02–84.60%), specificity of 73.58% (95% CI: 60.42–83.56%), PPV of 65.85% (95% CI: 50.55–78.44%), NPV of 79.59% (95% CI: 66.36–88.52%), and overall accuracy of 73.33% (95% CI: 63.38–81.38%). Following POCUS implementation, the annualized hospital admission rate for pediatric facial odontogenic infections decreased from 60.0 to 19.5 admissions/year; rate ratio (RR) = 0.33 (95% CI: 0.25–0.42), *p* < 0.001 (Poisson regression with log-offset for period length). Conclusions: Handheld POCUS, operated by non-specialist clinicians after a defined training protocol, was associated with a lower annualized admission rate and demonstrated moderate diagnostic accuracy. Its adoption was associated with a notable reduction in hospitalizations, suggesting its potential for alleviating ED overcrowding, reducing healthcare costs, and minimizing pediatric stress. Wider adoption, supported by standardized training, could enhance healthcare efficiency and quality in managing this common pediatric condition.

## 1. Introduction

Pediatric odontogenic infections, mainly caused by dental caries, are a common reason for emergency department (ED) visits. In the U.S., pediatric odontogenic infections make up a notable portion of ED visits. While exact numbers vary, studies show that dental disorders, including infections, account for about 1.4% of all ED visits each year [1]. Specifically, among pediatric dental-related visits, periapical abscesses make up to 47% of cases seen in pediatric EDs. These infections often result from untreated dental caries.

In Israel, pediatric odontogenic infections are also a significant part of dental emergencies. A study on pediatric dental visits to an emergency clinic found that pulpal inflammation—an early stage of odontogenic infection—was the main complaint in 43% of cases. Additionally, dentoalveolar abscesses are a major cause of hospitalization for dental problems in children; one study recorded 1413 pediatric admissions over six years within a major Israeli health system.

Globally, odontogenic infections in children remain common, mainly due to the worldwide burden of untreated dental caries. Although comprehensive global data on ED visit rates are limited, regional studies show that odontogenic infections are among the leading causes of pediatric dental emergencies. For instance, in Germany, they constituted 9.2% of dental emergency visits at a major outpatient center. These infections are frequently associated with pain, school absenteeism, and, in severe cases, hospitalization.

If not managed promptly and appropriately, these infections can escalate from localized dental abscesses to more severe conditions such as facial cellulitis, deep neck space infections [2], Ludwig’s angina with potential airway compromise, and, in rare instances, life-threatening systemic sepsis or intracranial spread [3,4].

A critical decision point in managing acute pediatric odontogenic infections is the differentiation between cellulitis—a diffuse inflammation of soft tissues typically responsive to antibiotic therapy—and a loculated abscess, which generally necessitates surgical incision and drainage (I&D) for resolution. This distinction can be particularly challenging in pediatric patients due to their often-limited ability to precisely describe symptoms, cooperate with physical examinations, or tolerate discomfort, thereby complicating clinical assessment [5,6]. While experienced oral and maxillofacial surgeons (OMFSs) or pediatric dental specialists may possess heightened clinical acumen for detecting abscesses, general pediatricians, emergency physicians, and junior clinicians—who are often the first point of contact—may find this differentiation difficult [7]. This diagnostic uncertainty can contribute to delays in appropriate treatment, increased ED utilization, potentially avoidable hospital admissions, prolonged patient suffering, and, consequently, increased healthcare expenditures [8].

Traditional advanced imaging techniques, such as computed tomography (CT) and magnetic resonance imaging (MRI), offer high accuracy in detecting abscesses and evaluating the extent of infection, particularly in complex cases or when deep space involvement is suspected. On CT scans, cellulitis typically appears as diffuse, poorly defined soft tissue swelling with increased density, without a distinct fluid collection. In contrast, abscesses often present as well-defined, low-attenuation lesions with rim enhancement and may contain internal gas bubbles. MRI findings include diffuse soft tissue edema and enhancement in cellulitis, while abscesses appear as T2-hyperintense and T1-hypointense fluid collections with rim enhancement after contrast administration. Although these imaging features help differentiate between the conditions, they are less accessible in urgent pediatric settings [9]. However, these modalities have significant drawbacks in the pediatric population. CT scans involve ionizing radiation, which raises concerns about cumulative exposure in children. In contrast, MRI scans are costly, time-consuming, and often require sedation or general anesthesia to ensure high-quality images in younger, uncooperative patients [10]. Additionally, neither CT nor MRI is usually available for immediate bedside use in most emergency departments or outpatient clinic settings.

This scenario underscores a gap in the availability of a fast, precise, non-invasive, radiation-free, and easily accessible diagnostic tool suitable for point-of-care use. While portable cart-based ultrasound devices have been a mainstay in emergency and outpatient care, the advent of compact, smartphone-connected handheld ultrasound devices marks a significant innovation. These devices offer comparable imaging for superficial structures, combined with greater affordability, ease of use, and accessibility. Their pocket-sized design helps reduce logistical barriers, allowing for broader adoption across diverse clinical settings. This democratization of ultrasound technology enables frontline providers—often without traditional radiology support—to incorporate imaging directly into their clinical workflows. Handheld point-of-care ultrasound (POCUS) has thus emerged as a promising technology that satisfies many of these needs [11,12]. Its portability, lower cost, absence of ionizing radiation, and capability for real-time dynamic imaging make it an appealing solution for various diagnostic challenges in pediatric emergency medicine and outpatient environments [6,13]. Past studies, including systematic reviews and meta-analyses, confirm POCUS’s high diagnostic accuracy in distinguishing cellulitis from abscess in skin and soft tissue infections (SSTIs) in both adults and children, often outperforming clinical examination alone [11]. Foundational research also indicates that POCUS findings can significantly impact clinical management plans for SSTIs in many patients [14,15].

Despite the growing body of evidence for general SSTIs, there is a relative paucity of data specifically addressing the utility of POCUS for pediatric facial odontogenic infections, particularly when performed by non-specialist clinicians (e.g., pediatricians, emergency physicians, or general dental practitioners) who have undergone a focused training protocol. Facial anatomy is complex, and the sonographic appearance of infections can be nuanced. Therefore, understanding the performance characteristics of POCUS in this specific context and by this operator group is crucial for its broader implementation.

Integrating easily accessible and interpretable handheld POCUS into the routine assessment of pediatric odontogenic infections could revolutionize current clinical pathways. If proven accurate, it could empower frontline clinicians to make more confident and timely decisions, leading to expedited incision and drainage (I&D) for abscesses, more conservative and targeted antibiotic use for cellulitis, reduced unnecessary referrals for advanced imaging, and decreased avoidable hospital admissions [16]. This, in turn, could alleviate the burden on EDs, reduce patient and family stress, minimize exposure to radiation and sedation, and enhance the overall quality and efficiency of care [17].

Novelty and Aim. Rather than assessing cart-based systems or expert sonographers, this study evaluates a handheld device used by novice non-radiologist operators (OMFS residents following focused training) for a specific pediatric indication—differentiating cellulitis from abscess at the point of care—and examines an institutional outcome using annualized admission rates with an explicit statistical framework. This pragmatic perspective complements prior work and addresses an implementation gap in everyday pediatric practice.

## 2. Materials and Methods

*Study Design and Setting*

Study Design and Setting. This was a prospective observational study assessing diagnostic accuracy, conducted from March 2021 to December 2022 at Rambam Health Care Campus. A total of 111 pediatric patients (ages 1–17) with maxillofacial odontogenic infections were enrolled and evaluated with handheld ultrasound. Based on the 2022 study findings, handheld ultrasound was subsequently integrated into routine practice for suspected pediatric odontogenic infections in the emergency department.

Admissions Cohort Definition and Comparative Analysis. We queried institutional records for pediatric presentations with superficial odontogenic infections in two windows: pre-implementation (Jan 2017–December 2021) and post-implementation (Jan 2022–April 2025). Inclusion: ages 1–17 years, ED/OMFS presentations consistent with odontogenic infection; exclusion: deep-space infections and non-odontogenic facial infections. When documented, admission-time diagnosis was abstracted as cellulitis or abscess; otherwise coded as not categorized. Treatment during admission (antibiotics alone, extraction, I&D) was abstracted when available. We compared annualized admission rates using Poisson regression (log link) with period length as an offset, reporting rate ratios (RRs) with 95% CIs and *p*-values from likelihood-ratio tests. Because retrospective documentation was variably complete, estimating “unnecessary hospitalization” was not reliable and is treated, if mentioned, as exploratory only.

Ethical Considerations: The study protocol was approved by the Institutional Review Board (IRB) of Rambam Health Care Campus (Helsinki Committee, RMB-0404-22). The study adhered to the principles of the Declaration of Helsinki. Written informed consent was obtained from a parent or legal guardian for all children aged 1–17 years prior to study procedures. Additionally, assent was obtained from capable children within this age range (typically from 7 or 8 years old and older, by IRB guidelines) who understood the study’s purpose and procedure. The institutional review board approved the study as minimal risk, recognizing POCUS as an extension of the clinical examination. The protocol did not mandate pre-study supervised quotas, which we acknowledge as a methodological limitation, No procedure was scheduled for research purposes; during the first 7 days, any extraction or I&D occurred only if clinically indicated as part of routine care.

Patient Selection and Enrollment

A convenience sample of pediatric patients was prospectively enrolled. Patients were eligible for inclusion if they were aged 1 to 17 years and presented with clinical signs and symptoms suggestive of an acute maxillofacial odontogenic infection. These signs included, but were not limited to, localized or diffuse facial swelling, pain, erythema, warmth, trismus, fever, and a history or clinical finding suggestive of a dental origin (e.g., carious tooth, recent dental procedure, gingival swelling). Exclusion criteria were:

1. Infections are clearly of non-odontogenic origin.
2. Superficial skin pustules without deeper involvement or suspected dental source.
3. Patients with known significant hematological or immunological disorders.
4. Lack of consent from the parent/guardian or assent from the child when appropriate.
5. Incomplete critical data that would preclude definitive classification by the reference standard.
6. Patients who had undergone surgical intervention for the current infectious episode prior to POCUS assessment at our institution.

The treating clinicians identified eligible patients. If eligibility criteria were met, a research team member approached the parent/guardian to explain the study and obtain informed consent.

Clinical Assessment Protocol:

All enrolled patients underwent a standardized clinical examination by an OMFS resident or a pediatric emergency physician prior to POCUS. This included detailed medical/dental history, systemic evaluation, and extraoral/intraoral examinations to identify potential odontogenic sources and characterize the infection. A presumptive diagnosis and preliminary management plan were documented.

Diagnostic and Treatment Protocol. All children underwent standardized clinical assessment (vitals, airway, pain, swelling character, trismus, dentition). When cellulitis was suspected and no airway compromise criteria were present, clinicians initiated conservative management with oral antibiotics per institutional protocol and analgesia, with tooth extraction performed when the infected primary tooth was deemed the likely source and extraction was clinically indicated. When abscess was clinically and/or sonographically evident, incision and drainage (I&D) was performed in the clinic, ED procedure room, or operating room at the clinician’s discretion. No procedure was scheduled for research purposes; during the first 7 days after enrollment, interventions were undertaken only if clinically indicated as part of routine care. Follow-up occurred within 7 days to document resolution, need for procedure, or change in therapy.

POCUS Training Protocol for Non-Specialist Clinicians

Clinicians designated as POCUS operators for this study were OMFS residents spanning Years 1 to 5. None of these residents had previous ultrasound experience before beginning training with the Philips Lumify handheld device. The training, developed and led by experienced ultrasound practitioners, including attending physicians and radiologists, included an intensive didactic component lasting approximately 10 h. This structured program comprised:

4 h of lectures covering ultrasound physics, knobology, and sonoanatomy of the head and neck.

2 h of video-based instruction on soft tissue infections, including visual recognition of abscess and cellulitis features.

2 h of group-based review of anonymized clinical cases with discussion of diagnostic pitfalls.

2 h of practical hands-on sessions using the handheld device on healthy volunteers and phantom models to build probe handling, image optimization, and scanning technique skills.

The program focused on systematic scanning protocols, identifying key landmarks, and dynamic assessment using probe compression. Although formal supervised scanning of patients was not performed prior to the study, trainees became familiar with image acquisition and interpretation before enrollment.

The training was designed for pragmatic feasibility in a pediatric workflow and was not intended to establish universal competency. This study evaluates the achievable performance of novice operators after focused preparation; future work should define competency-based curricula and establish supervised scanning requirements.

**Figure 1:**
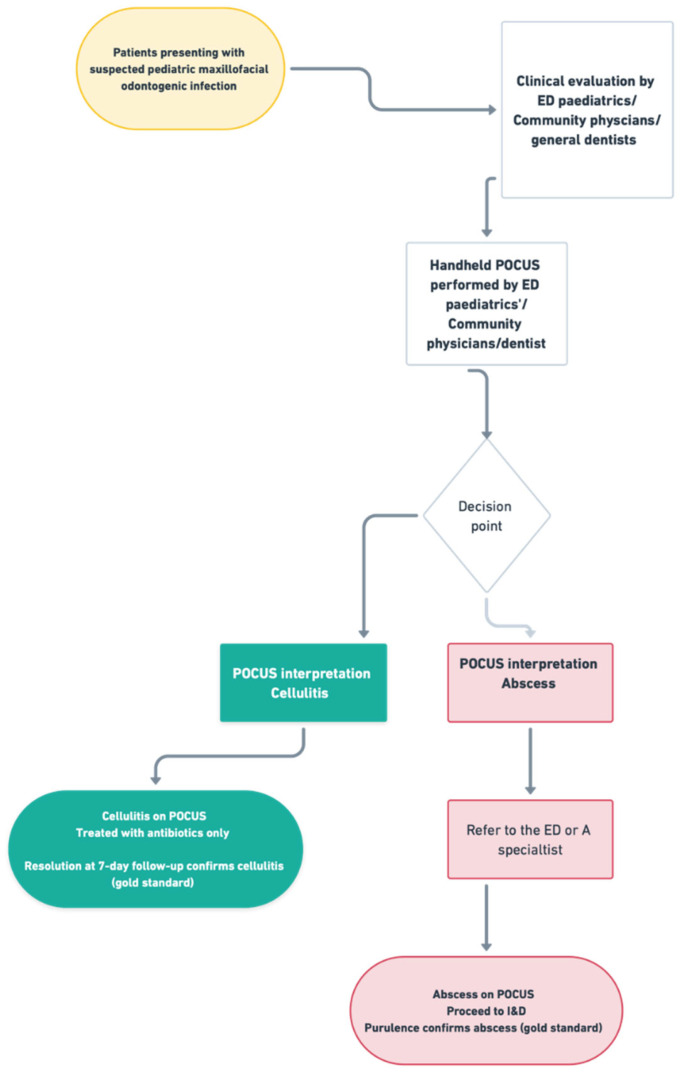
Handheld POCUS examination protocol. Systematic scanning of the affected region in two orthogonal planes, with dynamic compression assessment.

**Figure 2:**
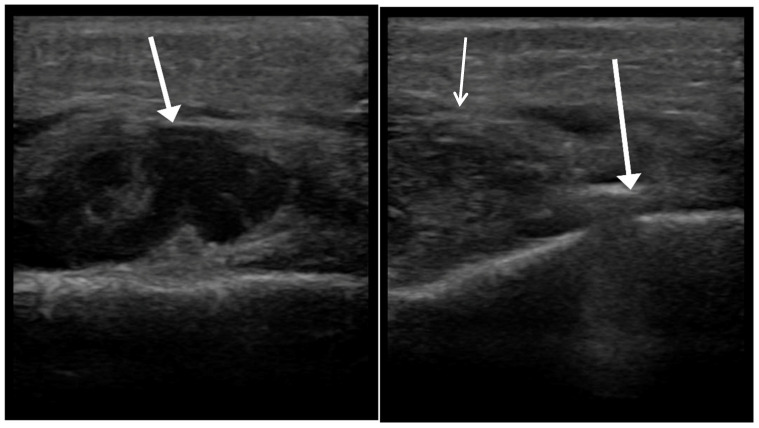
Handheld ultrasound imaging. **Left**: assessment of suspected abscess (arrow). **Right**: intraoperative ultrasound guidance confirming abscess location adjacent to periosteal elevator (arrow).

**Figure 3:**
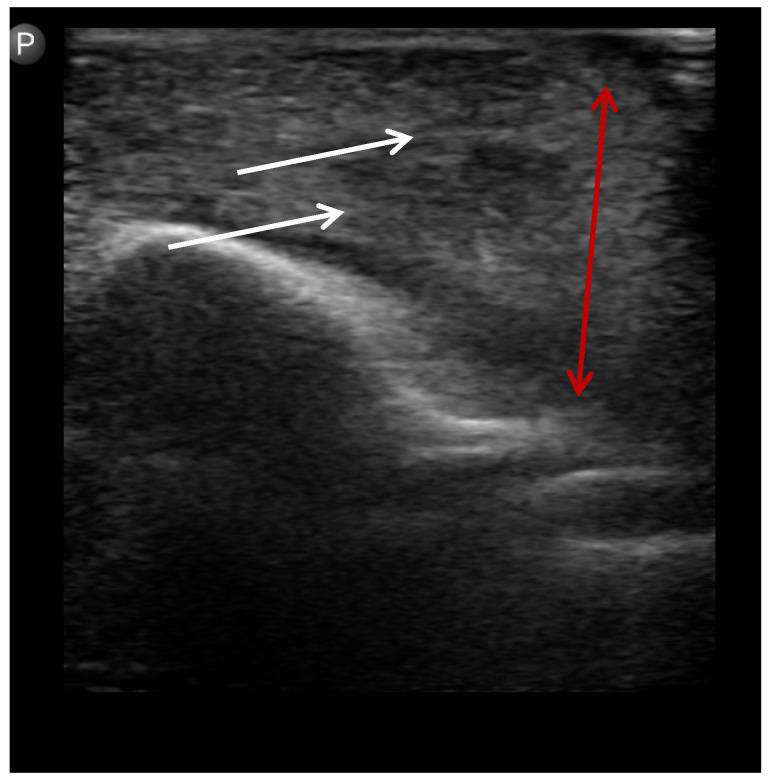
Ultrasound image showing cellulitis. White arrows: diffuse fascial thickening without a discrete collection. Red arrow: infiltration consistent with cellulitis.

POCUS Examination Protocol:

After conducting a clinical assessment and obtaining consent, POCUS was performed at the patient’s bedside or in the clinic by a trained OMFS resident using a Philips Lumify handheld ultrasound system (Philips Healthcare, Amsterdam, The Netherlands), equipped with a high-frequency linear array transducer (L12-4 MHz). The examination involved standard patient positioning, optimizing transducer settings for superficial soft tissues, and systematically scanning the affected area in at least two orthogonal planes, including a dynamic assessment with gentle transducer pressure. Basic B-mode imaging was the primary modality used, with the routine use of advanced Doppler modes not being required for this application. Representative images and/or short video clips were saved directly to the dedicated smartphone connected to the Lumify device. This phone was kept secure, with no external internet access except through the secure internal hospital network when needed, and remained in the residents’ possession. The operator documented their interpretation (abscess, cellulitis, or ambiguous), location, and estimated three-dimensional size of any collection on a standardized reporting form immediately after the scan. POCUS operators were not blinded to the initial clinical assessment findings, as POCUS was considered an extension of the clinical examination. Accordingly, this study does not measure the incremental value of POCUS over clinical examination alone; it reports performance within an integrated, real-world workflow. No specific blinding measures were used regarding the final reference standard results during POCUS interpretation (Figure 1, Figure 2 and Figure 3).

Reference Standard Determination

All patients diagnosed with abscesses underwent surgical intervention (incision and drainage) either in the emergency department or the operating room. No abscess case was managed conservatively. Patients diagnosed with cellulitis were managed with antibiotics alone and monitored via clinical follow-up to confirm resolution.

Abscess Confirmation: Presence of purulent material expressed or aspirated during I&D.

Cellulitis Confirmation: Complete resolution of signs and symptoms with antibiotic therapy alone, without the need for drainage, assessed during a 7-day follow-up after the ED visit. Resolution was determined through follow-up clinical examinations and/or structured phone interviews with the parent or guardian, specifically inquiring about the resolution of swelling and redness, as well as the return to facial symmetry.

Data Collection: Standardized data collection forms recorded demographics, history, clinical signs, POCUS findings, I&D details, therapy, admission data, LOS, and follow-up information. Data were entered into Microsoft Excel.

Note on Imaging: This study did not utilize advanced imaging methods, such as CT and MRI, as diagnostic standards for two primary reasons. Firstly, the infections studied were superficial odontogenic abscesses and cellulitis, not deep space infections, which are typically diagnosed through clinical assessment. Secondly, MRI was rarely available at our institution and not generally accessible for urgent diagnosis in pediatric dental infections. Although CT is effective for detecting abscesses, it was avoided due to concerns about the potential for ionizing radiation exposure in children. Instead, the reference standard included surgical drainage for abscesses or complete clinical resolution with antibiotics for cellulitis, aligning with a practical and ethical approach suitable for pediatric patients. Nonetheless, CT and MRI remain the most accurate imaging tools for evaluating deep or complex infections when needed.

We acknowledge that this asymmetric reference standard may introduce verification bias; its implications are detailed in the Discussion.

Outcome Measures

Primary Outcome: Diagnostic accuracy of POCUS, including sensitivity, specificity, PPV, NPV, and overall accuracy, with their 95% CIs.

Secondary Outcome: Institutional annualized hospital admission rates for pediatric odontogenic infections (pre-POCUS: January 2017–December 2021; post-POCUS: January 2022–April 2025), analyzed as admissions/year, and length of stay (LOS) for admitted patients from the study cohort. No other secondary outcomes were formally analyzed for this report.

Statistical Analysis Data were analyzed using SPSS Statistics version 27 (IBM Corp., Armonk, NY, USA; SPSS defined at first use). Descriptive statistics (mean ± standard deviation [SD] or median and interquartile range [IQR] for continuous variables, and frequencies and percentages for categorical variables) were used to summarize the data. Diagnostic performance metrics and their 95% CIs (Clopper-Pearson method) were calculated from 2 × 2 contingency tables. Continuous variables (e.g., LOS) were compared using independent-sample *t*-tests if data were normally distributed (assessed by the Shapiro–Wilk test or visual inspection of histograms) or Mann–Whitney U tests if not. Categorical variables were compared using chi-square tests or Fisher’s exact test based on expected cell counts. For institutional admissions, we compared annualized admission rates (admissions per year) before vs. after POCUS implementation using Poisson regression (log link) with period length as an offset. We report rate ratios (RRs) with 95% Wald confidence intervals, with *p*-values from likelihood-ratio tests. As a sensitivity check, we corroborated the results with a chi-square test on period totals. A *p*-value < 0.05 was considered statistically significant. No adjustments were made for multiple comparisons. No multiplicity correction was applied to sensitivity, specificity, PPV, and NPV because these co-primary descriptive metrics arise from the same contingency table and jointly characterize diagnostic performance; inferential testing was limited to prespecified comparisons.

## 3. Results

### 3.1. Patient Characteristics and Enrollment

From March 2021 to December 2022, 111 pediatric patients were prospectively enrolled. Twenty-one patients were excluded for the following reasons: (1) ambiguous POCUS findings with no definitive imaging or follow-up to determine diagnosis (*n* = 14), defined as sonographic results that lacked sufficient clarity to confidently identify either abscess (e.g., hypoechoic, loculated area with posterior enhancement) or cellulitis (e.g., diffuse fascial thickening without a discrete collection); (2) missing critical age data (*n* = 1); and (3) Similarly, six patients had indeterminate outcomes either due to incomplete follow-up data or ambiguous treatment response, preventing assignment to a definitive diagnostic category per our reference standard definitions (*n* = 6). The final cohort for diagnostic accuracy analysis consisted of 90 patients. The mean age of these 90 patients was 7.8 ± 3.74 years (range: 1–17 years); 52 (58.0%) were male. The most common suspected dental sources were carious primary molars (*n* = 60, 66.7%) and carious permanent molars (*n* = 30, 33.3%).

### 3.2. Ultrasound Diagnostic Performance

**Figure 4:**
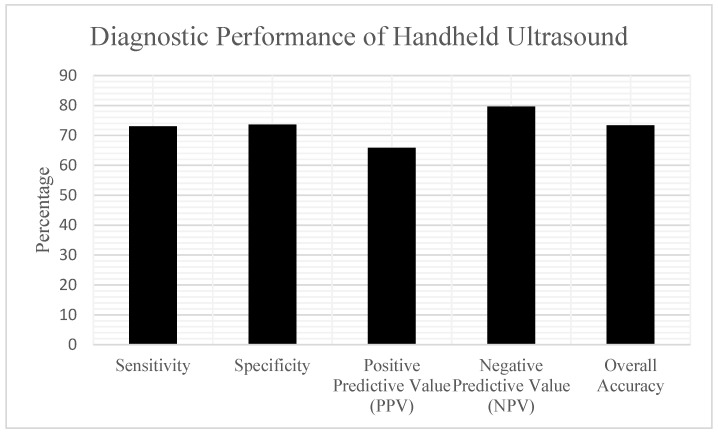
Bar graph depicting sensitivity, specificity, positive predictive value (PPV), negative predictive value (NPV), and overall diagnostic accuracy for handheld ultrasound in diagnosing pediatric odontogenic infections.

According to the reference standard determination, 37 patients (41.1%) in the analysis cohort had a confirmed abscess, while 53 patients (58.9%) had cellulitis. The handheld POCUS examinations yielded the following results when compared to the reference standard (Table 1) (Figure 4):

True Positives (TP-POCUS identified abscess, abscess confirmed): 27;

False Positives (FP-POCUS identified abscess, cellulitis confirmed): 14;

True Negatives (TN-POCUS identified cellulitis, cellulitis confirmed): 39;

False Negatives (FN-POCUS identified cellulitis, abscess confirmed): 10.

Based on these findings, the diagnostic performance characteristics of handheld POCUS were:

Sensitivity: 72.97% (27/37) (95% CI: 57.02–84.60%);

Specificity: 73.58% (39/53) (95% CI: 60.42–83.56%);

Positive Predictive Value (PPV): 65.85% (27/41) (95% CI: 50.55–78.44%);

Negative Predictive Value (NPV): 79.59% (39/49) (95% CI: 66.36–88.52%).

Overall Diagnostic Accuracy was 73.33% (66 out of 90), with a 95% CI of 63.38 to 81.38%. Of the 14 false positive cases—where POCUS indicated abscess but clinical findings supported cellulitis—most showed sonographic features like small hypoechoic areas that either did not result in purulence during surgical exploration or resolved with antibiotics alone. All these cases were ultimately diagnosed as cellulitis. Of the 10 false negative cases—where POCUS suggested cellulitis but an abscess was later confirmed—several patterns were observed. Some collections were very small or deep, obscured by overlying edema and not readily apparent on bedside scanning. In other cases, limited cooperation and discomfort during probe compression restricted image acquisition. All 10 patients subsequently underwent incision and drainage, with purulent material confirming abscess. Importantly, none progressed to airway compromise or deep space infection.

Out of 37 patients diagnosed with abscess, all received surgical incision and drainage, with purulent material confirming the diagnosis. Conversely, the 53 patients with cellulitis were treated solely with antibiotics and achieved full clinical resolution without requiring drainage or advanced imaging.

### 3.3. Clinical Outcomes

Within the analyzed cohort of 90 patients, 60 (66.7%) were admitted to the hospital for management. For these 60 admitted patients, the mean LOS was 1.67 ± 1.24 days. There was no statistically significant difference in LOS for these admitted patients based on their POCUS result (POCUS Indicated abscess vs. POCUS Indicated Cellulitis) when other clinical criteria for admission were met (*p* > 0.05, Independent-sample *t*-test).

### 3.4. Impact on Hospital Admissions

**Figure 5:**
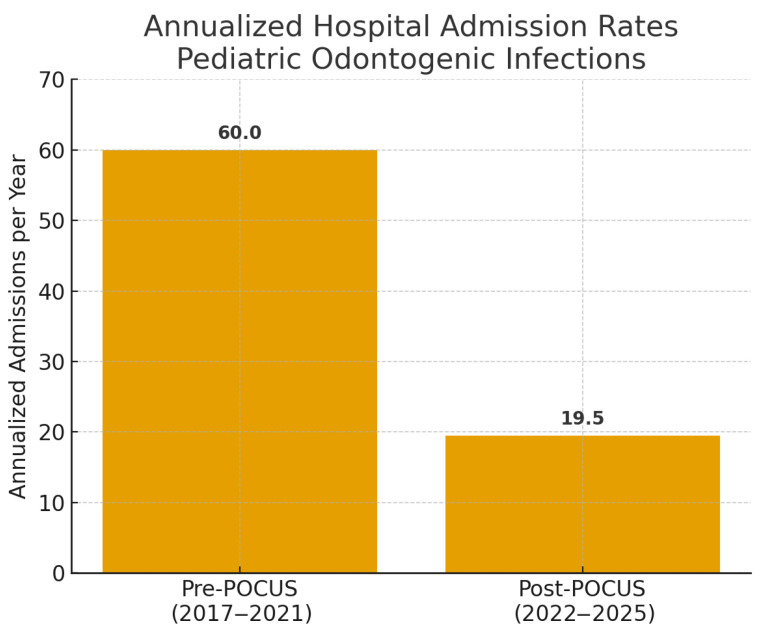
Annualized hospital admission rates for pediatric odontogenic infections before and after handheld POCUS implementation. Pre-POCUS (2017–2021): 60.0 admissions/year (300 total over 5 years). Post-POCUS (January 2022–April 2025): 19.5 admissions/year (65 total over 3.33 years). A 78.3% reduction was observed (RR = 0.33, 95% CI: 0.25–0.42, *p* < 0.001).

Annualized institutional hospital admissions for pediatric odontogenic infections decreased from 60.0/year in the pre-POCUS period (2017–2021; 300 total admissions) to 19.5/year in the post-POCUS period (January 2022–April 2025; 65 total admissions). This reduction was statistically significant, corresponding to a rate ratio (RR) of 0.33 (95% CI: 0.25–0.42, *p* < 0.001, Poisson regression) Figure 5).

This comparison is associational and may be influenced by time-varying confounders (e.g., utilization patterns, staffing, referral or triage shifts), When diagnosis at admission was documented, cases were classified as cellulitis or abscess; missing documentation was recorded as not categorized. Treatment modality during admission was abstracted when available.

## 4. Discussion

This prospective study demonstrates that handheld POCUS, employed by OMFS residents with no prior ultrasound experience after a focused didactic training program, is a moderately accurate tool for differentiating abscess from cellulitis in pediatric acute maxillofacial odontogenic infections. Furthermore, its systematic implementation was associated with a clinically and statistically significant reduction in annualized hospital admission rates at our institution: admissions decreased from 60.0 to 19.5 per year; rate ratio (RR) = 0.33 (95% CI: 0.25–0.42), *p* < 0.001 (Poisson regression with log-offset for period length). These findings support the potential role of handheld POCUS in pediatric emergency and dental care pathways while avoiding causal inference.

Diagnostic Performance of POCUS in Context.

Our study found a POCUS sensitivity of 73.0% and a specificity of 73.6% for detecting abscesses. A key observation is that these moderate yet clinically useful metrics were achieved by OMFS residents who were novices to ultrasound, following approximately 10 h of primarily didactic training supplemented with practical demonstrations and case reviews for this focused application. This level of accuracy, particularly the NPV of 79.6%, may support outpatient management when combined with thorough clinical evaluation, potentially avoiding unnecessary interventions. While the PPV of 65.9% indicates that a POCUS finding suggestive of an abscess warrants careful clinical correlation, the overall performance is noteworthy given the operators’ initial experience level. An overall accuracy near 73% with an NPV around 80% may be clinically useful for ruling out abscess in lower-risk cases when aligned with clinical assessment; it is not intended for independent, stand-alone decision-making in pediatric emergency care. When we compare our findings with the broader POCUS literature on SSTIs, our diagnostic accuracy metrics may seem somewhat lower than the high pooled sensitivities (often exceeding 90%) observed in meta-analyses, such as those by Gottlieb et al. [11]. This discrepancy is likely due to several factors. First, many studies included in these meta-analyses feature POCUS conducted by highly skilled sonographers or emergency physicians trained in POCUS fellowships, frequently using advanced cart-based ultrasound systems. Second, the anatomy of the face and neck is inherently more intricate than that of the limbs or trunk, making it more challenging to identify small or deep collections. Third, our study specifically focused on OMFS residents who were new to POCUS, utilizing a handheld device after a concentrated training that emphasized didactic learning for a particular binary question. The fact that “approximately 10 h” of such training could lead to this level of diagnostic capability is encouraging. It suggests that, for this purpose, POCUS is a skill that can be readily learned. This is consistent with the broader understanding that POCUS for certain targeted applications can be effectively learned through practical training [6,13], making it a valuable complement rather than a flawless standalone test, assisting clinicians more effectively than relying on clinical examination alone [7].

Verification bias can arise due to an uneven reference standard. In this study, abscesses were confirmed through incision and drainage (I&D) with direct evidence of purulence, while cellulitis was defined by symptom resolution on antibiotics within 7 days of clinical or phone follow-up. This difference can lead to verification bias: small or early abscesses may improve temporarily on antibiotics and be mislabeled as “cellulitis,” hiding the true abscesses. Such mislabeling can alter diagnostic estimates, likely underestimating sensitivity (true abscesses not detected by the reference standard), reducing PPV, and inflating specificity and NPV if mislabeled abscesses are counted as true negatives. These effects highlight the need to interpret handheld POCUS in a clinical context and reassess symptoms serially when they persist or return despite antibiotics. To mitigate these issues, consider (i) standardizing return and recall criteria for re-examination and repeat POCUS, (ii) conducting in-person follow-ups beyond 7 days for initial improvement that may be temporary, and (iii) using a composite reference standard (I&D, antibiotic failure, or confirmatory imaging when indicated) adjudicated by a blinded panel in future studies.

Jaw-specific considerations. Anatomical spread patterns and sonographic windows differ between maxillary and mandibular infections, which may influence both false-negative and false-positive rates (e.g., thin cortical plates and canine fossa in the maxilla vs. thicker mandibular cortex and subperiosteal spread). Our study did not stratify performance by jaw, and future work should examine maxilla- vs. mandible-specific accuracy and workflow.

Clinical Impact and Transformation of Care Pathways.

This marked reduction in admissions following POCUS integration highlights its potential to shift management toward outpatient care, reduce unnecessary hospitalizations, and alleviate ED burden. Here, we report a single-center prospective evaluation from Israel to quantify handheld POCUS performance and to describe institutional annualized admission patterns after adoption. The scope is intentionally contextual rather than global in nature. By enabling non-specialist clinicians in these initial contact points to more confidently differentiate cellulitis from an abscess, POCUS may facilitate better triage decisions, particularly regarding the necessity of referral to an ED. This pattern is consistent with potential downstream benefits. Busy EDs may experience reduced burden from non-urgent odontogenic infection referrals, allowing them to focus on more critical cases. More importantly, the patient and family experience can be substantially improved. If a child’s condition can be safely and accurately identified as cellulitis amenable to oral antibiotics, an unnecessary and often traumatic ED visit and potential hospital admission may be avoided. Hospitalization itself can induce considerable stress in children and their parents, particularly for families with other children at home or those facing work and financial pressures. Minimizing these experiences by making definitive outpatient management more feasible is a crucial patient-centered outcome. Furthermore, by providing objective, real-time visualization, POCUS may help clinicians expedite appropriate consultation and definitive treatment (I&D) for confirmed abscesses, while also fostering more judicious use of antibiotics and potentially reducing reliance on CT scans, thereby minimizing radiation exposure [10].

With a PPV of 65.9%**,** approximately one-third of positive scans did not correspond to purulence at I&D. Potential consequences—such as unnecessary surgical exploration—underscore that POCUS should function as an adjunct to the clinical examination rather than a stand-alone arbiter. Positive studies should be correlated with exam findings before intervention to reduce overtreatment risk.

Implications for Training and Broader Adoption.

Our study suggests that training OMFS residents with no prior US experience to effectively use handheld POCUS for this specific purpose is feasible through a relatively short, practical, mainly didactic program. This is a key observation because it indicates that the obstacle to adopting POCUS for this common diagnostic challenge may be less significant than usually believed, especially with user-friendly handheld devices. This could facilitate wider dissemination of this skill beyond specialists to pediatricians, emergency physicians, pedodontists, and general dentists, who frequently encounter these infections. Such training may help them make better initial management and referral choices, positively impacting patient care and healthcare system efficiency. Handheld ultrasound devices have demonstrated high diagnostic reliability across various medical specialties. In obstetrics and gynecology, for example, studies have reported strong agreement with conventional ultrasound systems—as evidenced by high intraclass correlation coefficients (ICCs)—for assessments such as fetal biometry, postpartum uterine evaluation, and the diagnosis of postpartum urinary retention [18,19]. These findings support the feasibility of incorporating handheld ultrasound into clinical workflows with minimal but focused training, aligning with our study’s results in the context of pediatric odontogenic infections. These findings strengthen the case for the reliability of handheld ultrasound and support its wider adoption, especially given its portability, lack of ionizing radiation, and usefulness in point-of-care settings. However, while our approximately 10 h didactic and demonstration-based training resulted in moderate accuracy, the findings also implicitly support the importance of carefully structured training. Future efforts should focus on establishing optimal, standardized, yet practical training pathways for various non-specialist clinician groups. These pathways must strike a balance between thorough didactic learning and hands-on skills development—possibly through simulation, supervised scanning on a range of cases, and image review—and include quality assurance measures to ensure consistent and reliable use of POCUS.

Handheld ultrasound devices provide significant advantages such as portability, affordability, and user-friendliness at the point of care. These qualities may enable quick decision-making in outpatient and emergency environments, particularly by non-specialists. However, they often have lower image resolution, lack some advanced features like Doppler, and offer limited data storage compared to standard machines. Despite this, for specific exams—such as distinguishing cellulitis from abscess—they serve as a convenient, practical option that can aid accurate bedside diagnosis.

Strengths of the Study

This study’s strengths include its prospective design, focus on a common pediatric issue, utilization of modern handheld POCUS technology, and assessment of both diagnostic accuracy and a key clinical outcome—hospital admission rates. Clinicians valued the handheld ultrasound device’s practicality in pediatric emergencies, highlighting its portability, ease of operation, safety, and ability to provide real-time assessments. They reported that it enabled faster, more confident decisions, particularly among residents after brief training. This positive perception has contributed to the routine use of POCUS for pediatric odontogenic infections within our institute and department.

Technical and operator considerations. Handheld systems trade ultimate image resolution and feature depth for portability; advanced modes (e.g., Doppler) were not routinely used in this protocol. Performance is operator-dependent, particularly in small or uncomfortable children, and image windows may be limited by pain or cooperation. Handheld POCUS is unsuitable for deep space infections, which typically require CT/MRI and specialist management [10].

Limitations

This study has several limitations.

Single-center design. Findings from a tertiary academic medical center may not be generalizable to other settings.

Operator training and learning curve. POCUS examinations were performed by OMFS residents who were novices to ultrasound at the study outset. Although training was focused (~10 h), the absence of pre-study supervised scanning quotas or formal competency validation may have introduced a learning-curve effect that influenced accuracy.

Lack of blinding/incremental value. Operators were not blinded to clinical findings; the study, therefore, does not isolate the incremental diagnostic value of POCUS over clinical examination and is susceptible to bias.

Asymmetric reference standard (verification bias). Abscesses required confirmation via I&D with purulence, while cellulitis was diagnosed based on clinical or telephone follow-up of symptom resolution at 7 days. This discrepancy can lead to over-diagnosing cellulitis in small, early, or improving abscesses, causing misclassification and biased performance metrics (likely lower sensitivity/PPV and higher specificity/NPV). Although return precautions and serial reassessment were provided, a longer standardized in-person follow-up and predefined re-scan triggers would reduce bias. Future studies should adopt a composite reference standard with blinded adjudication.

Scope of infections. The cohort comprised superficial pediatric odontogenic infections (commonly from infected primary teeth, e.g., vestibular/buccal space processes). Deep-space infections (e.g., parapharyngeal, retropharyngeal) were excluded and typically require CT/MRI and specialist management; handheld ultrasound is not suitable for evaluating these entities.

Ambiguous cases excluded. Fourteen cases (12.6% of 111) with inconclusive sonographic features and no definitive reference outcome were excluded from the primary accuracy analysis. Excluding such cases can inflate or deflate performance estimates; future work should prospectively track uncertain cases to resolution.

Admissions analysis—observational comparison. The pre/post admissions evaluation was a retrospective institutional analysis (pre-implementation 2017–2021 vs. post-implementation January 2022–April 2025). Documentation was variably complete, and the comparison is associational, potentially affected by time-varying confounders (e.g., utilization patterns, staffing, referral/triage shifts).

Device/technical constraints. Handheld systems trade ultimate resolution and advanced modalities (e.g., Doppler not routinely used here) for portability; image acquisition can also be limited by pediatric cooperation and discomfort.

A key limitation is the asymmetry in our reference standard: abscesses were confirmed by I&D, while cellulitis was defined by clinical resolution with antibiotics. This creates potential verification bias. False negatives (abscesses initially misclassified as cellulitis) illustrate this conceptual limitation, as small or early abscesses may improve transiently on antibiotics before later requiring drainage.

Future studies should include multicenter designs with blinded interpretation, standardized competency-based training and supervised scanning, jaw-specific analyses (maxilla vs. mandible), prospective follow-up of ambiguous cases, and robust adjustment for temporal confounding in systems-level outcomes.

Future Research Directions:

Building on these findings, future research should focus on multicenter studies to confirm the generalizability of these results. Developing and rigorously evaluating standardized, competency-based POCUS training curricula for pediatric odontogenic infections—balancing practical needs with quality assurance—is essential. Comparative studies assessing different POCUS devices or exploring advanced ultrasound techniques (e.g., elastography) or AI decision-support tools used with POCUS could further improve diagnostic capabilities. Long-term studies examining patient outcomes over time and comprehensive cost-effectiveness analyses will offer a clearer understanding of POCUS’s value in this area.

## 5. Conclusions

Handheld point-of-care ultrasound, used by OMFS residents following approximately 10 h of structured training, showed moderate diagnostic accuracy for distinguishing abscess from cellulitis in children with acute maxillofacial odontogenic infections. At our institution, POCUS use was associated with a lower annualized admission rate, supporting its potential role in improving care efficiency; causal effects require confirmation in multicenter studies. While further efforts are needed to standardize training and validate these findings, this study supports broader adoption and integration of POCUS among various frontline pediatric and dental care providers, recognizing it as a valuable aid in managing this common and often complex pediatric condition.

## Figures and Tables

**Table 1:** Confusion matrix showing the relationship between ultrasound-predicted outcomes and surgical gold-standard confirmation. True positives (TP), false positives (FP), true negatives (TN), and false negatives (FN) are shown. Sensitivity = 72.97%, Specificity = 73.58%, PPV = 65.85%, NPV = 79.59%, Accuracy = 73.33%.

	Reference Standard Abscess (+)	Reference Standard Cellulitis (−)	Total
Ultrasound Predicted Abscess (+)	27 (TP)	14 (FP)	41
Ultrasound Predicted Cellulitis (−)	10 (FN)	39 (TN)	49
Total	37	53	90

## Data Availability

The raw data supporting the conclusions of this article will be made available by the authors on request.
